# Effect of asymptomatic transmission and emergence time on multi-strain viral disease severity

**DOI:** 10.1371/journal.pone.0269464

**Published:** 2022-10-07

**Authors:** Amir Reza Alizad-Rahvar, Mehdi Sadeghi

**Affiliations:** 1 School of Biological Sciences, Institute for Research in Fundamental Sciences (IPM), Tehran, Iran; 2 Department of Medical Genetics, National Institute for Genetic Engineering and Biotechnology, Tehran, Iran; Nanyang Technological University, SINGAPORE

## Abstract

In a viral epidemic, the emergence of a novel strain with increased transmissibility (larger value of basic reproduction number *R*_0_) sparks the fear that the increase in transmissibility is likely to lead to an increase in disease severity. It is required to investigate if a new, more contagious strain will be necessarily dominant in the population and resulting in more disease severity. In this paper, the impact of the asymptomatic transmission and the emergence time of a more transmissible variant of a multi-strain viral disease on the disease prevalence, disease severity, and the dominant variant in an epidemic was investigated by a proposed 2-strain epidemic model. The simulation results showed that considering only *R*_0_, is insufficient to predict the outcome of a new, more contagious strain in the population. A more transmissible strain with a high fraction of asymptomatic cases can substantially reduce the mortality rate. If the emergence time of the new strain is closer to the start of the epidemic, the new, more contagious variant has more chance to win the viral competition and be the dominant strain; otherwise, despite being more contagious, it cannot dominate previous strains. In conclusion, three factors of *R*_0_, the fraction of asymptomatic transmission, and the emergence time of the new strain are required to correctly determine the prevalence, disease severity, and the winner of the viral competition.

## Introduction

The emergence of the novel coronavirus strain in the UK, called SARS-CoV-2 VOC 202012/01 or B.1.1.7, was shocking because this novel variant could be up to about 70% more transmissible than pre-existing variants of SARS-CoV-2 [1]. This increased transmissibility can add between 0.4 and 0.7 to the basic reproduction number R_0_. This news sparked the fear that the increase in transmissibility is likely to lead to a large increase in hospitalization, intensive care unit (ICU) admission rate, and mortality. However, the studies about previous variants of SARS-CoV-2 showed that despite the rise of the lab-confirmed cases, the COVID-19 case fatality rate (CFR) declined, i.e., more transmissibility did not necessarily cause more severity [2, 3]. Other studies in the UK and England showed that besides increasing the COVID-19 cases, the hospitalization rate, the ICU admission rate, and the CFR declined [4, 5]. The preliminary explanation was the predominant shift towards positivity in younger age groups who have a better outcome. However, the analysis of German COVID-19 data [6], which was reported by age categories, showed that the COVID-19 CFR declined across all age groups [7]. Interestingly, the older groups drove the overall reduction in CFR.

The public health authorities need to pinpoint the cause of this decline in the fatality rate in order to decide how to react against the newly emerged viral strains. The decision to fight blindly against a novel strain because of its increased transmissibility is not necessarily the most comprehensive and effective solution. We need to take other factors along with the transmissibility into account in our decision-making.

The spread of COVID-19 is an iceberg with the invisible part of being the asymptomatic transmission [8]. The percent of asymptomatic cases who never experience COVID-19 symptoms remains uncertain and varies for different strains of SARS-CoV-2. For the original strain, from about 20% to 50% of infected people are reported to be asymptomatic [9–12]. In a study, 39% of children aged 6–13 years tested positive for COVID-19 with no symptoms [13]. Different studies reported an insignificant difference in the upper respiratory viral load between symptomatic and asymptomatic cases [9, 14]. [15] shows that the duration of viral shedding is similar in symptomatic and asymptomatic cases, which suggests the possibility of transmission during the asymptomatic period. Even a study found that asymptomatic patients had higher SARS-CoV-2 viral loads than symptomatic cases [16]. Consequently, the asymptomatic infected people could play a significant driver role in the community spread of COVID-19. The results of a study demonstrated that both *R*_0_ and the proportion of asymptomatic transmissions were the main factors in controlling an infectious disease outbreak [17].

In this study, we investigate the effect of the emergent viral strain on the total number of infected people and the illness severity by using epidemiological modeling. We will show that in an epidemic situation, the emergence time of the new strain and the relative *R*_0_ of the primary and the emergent strains determine the winner of the competition between two viral strains. Moreover, we will see that the disease severity and the cumulative mortality can be significantly influenced by the emergence time and the fraction of asymptomatic infectious cases of the emergent strain.

## Methods

**Fig 1:**
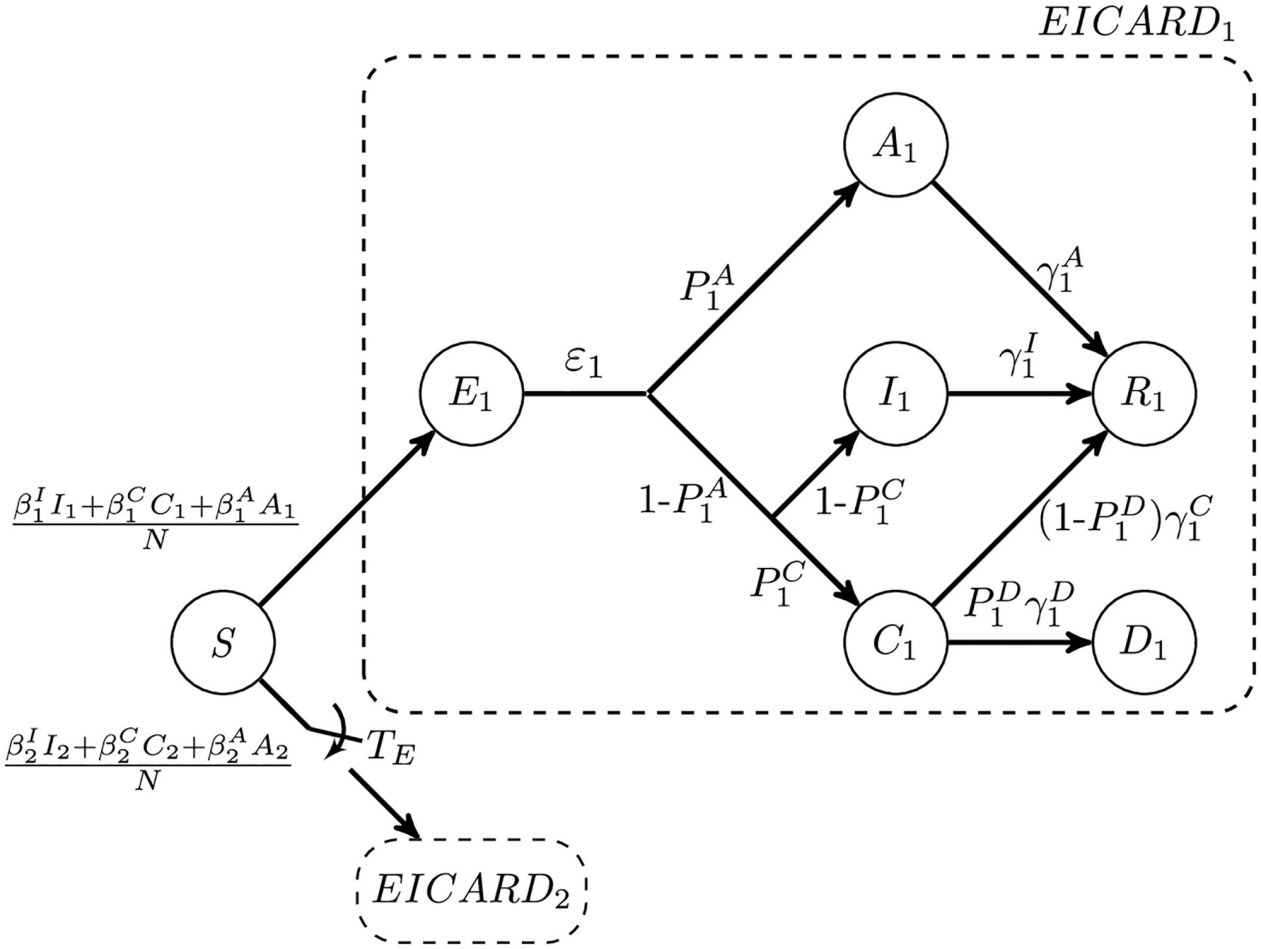
Schematic of the 2-SEICARD model. For simplicity, the lower branch, corresponding to the second strain, is not depicted, and it is denoted by *EICARD*_2_. The *EICARD*_2_ branch appears in the model for *t* ≥ *T*_*E*_.

For each viral strain, we use the extended version of the classic SEIR epidemic model, called the SEICARD model, consisting of susceptible (S), exposed (in the latent period) (E), symptomatic infected (I), critically infected (C), asymptomatic infected (A), recovered (R), and dead (D) people. By paralleling two SEICARD models, we develop a 2-strain model, called 2-SEICARD, that describes the existence and competition of two viral variants in the population (Fig 1). The index *s* = 1 or 2 in *E*_*s*_, *I*_*s*_, *C*_*s*_, *A*_*s*_, *R*_*s*_, and *D*_*s*_ represents the infectious strain in each group. It is assumed that the emergence time of the second strain is *T*_*E*_ days after the emergence time of the primary one, which is day 0. Moreover, we assume that there is no viral superinfection, i.e., the reinfection or co-infection between variants does not occur. In other words, the recovered individuals are cross-immunized and are immune to the new variants.

The parameters of the 2-SEICARD model are explained in Table 1. The ODE system of this 2-strain model is given by

$$\begin{array}{c} dS/dt=-\sum_{s=1}^{2}(\beta_{s}^{I}I_{s}+\beta_{s}^{C}C_{s}+\beta_{s}^{A}A_{s})S/N, \end{array}$$ (1a)

$$\begin{array}{c} dE_{s}/dt=(\beta_{s}^{I}I_{s}+\beta_{s}^{C}C_{s}+\beta_{s}^{A}A_{s})S/N-\varepsilon_{s}E_{s}, \end{array}$$ (1b)

$$\begin{array}{c} dI_{s}/dt=\left(1-P_{s}^{A}\right)\left(1-P_{s}^{C}\right)\varepsilon_{s}E_{s}-\gamma_{s}^{I}I_{s}, \end{array}$$ (1c)

$$\begin{array}{c} dC_{s}/dt=\left(1-P_{s}^{A}\right)P_{s}^{C}\varepsilon_{s}E_{s}-[\left(1-P_{s}^{D}\right)\gamma_{s}^{C}+P_{s}^{D}\gamma_{s}^{D}]C_{s}, \end{array}$$ (1d)

$$\begin{array}{c} dA_{s}/dt=P_{s}^{A}\varepsilon_{s}E_{s}-\gamma_{s}^{A}A_{s}, \end{array}$$ (1e)

$$\begin{array}{c} dR_{s}/dt=\gamma_{s}^{I}I_{s}+\left(1-P_{s}^{D}\right)\gamma_{s}^{C}C_{s}+\gamma_{s}^{A}A_{s}, \end{array}$$ (1f)

$$\begin{array}{c} dD_{s}/dt=P_{s}^{D}\gamma_{s}^{D}C_{s} \end{array}$$ (1g)

for *s* = 1 and 2. The total population is $N=S+\sum_{s=1}^{2}(E_{s}+I_{s}+C_{s}+A_{s}+R_{s}+D_{s})$. For simplicity, the natural birth and death rates are ignored in the model. To implement the emergence time *T*_*E*_, we set all parameters of the second strain to zero for *t* < *T*_*E*_.

**Table 1:** Explanation of the symbols of the 2-SEICARD model.

Symbol	Explanation	Value
*s*	Strain number	1, 2
*S*	Susceptible individuals	
*E*_*s*_	Exposed to strain *s* and still in the latent period	
*I*_*s*_	Symptomatic individuals infected with strain *s*	
*C*_*s*_	Critically infectious individuals infected with strain *s*	
*A*_*s*_	Asymptomatic individuals infected with strain *s*	
*R*_*s*_	Individuals recovered from strain *s*’ infection	
*D*_*s*_	Dead individuals infected with strain *s*	
*N*	Total number of individuals	10000
$P_{s}^{A}$	Fraction of asymptomatic individuals infected with strain *s*	0.1 (*s* = 1) 0.1, 0.2, 0.4 (*s* = 2)
$P_{s}^{C}$	Fraction of symptomatic individuals who are critically infected with strain *s*	0.1
$P_{s}^{D}$	Fraction of critically infected individuals who die from infection with strain *s*	0.05
$\beta_{s}^{I}$ , $\beta_{s}^{C}$, $\beta_{s}^{A}$	Infection rate of different outcomes of strain *s*	0.2 (*s* = 1) 0.13, 0.2, 0.27 (*s* = 2)
1/*ε*_*s*_	Average incubation period of strain *s*	5 days
1/$\gamma_{s}^{I}$, 1/$\gamma_{s}^{C}$, 1/$\gamma_{s}^{A}$, 1/$\gamma_{s}^{D}$	Average infection period of different outcomes of strain *s*	10 days
*T*_*E*_	Emergence time of strain 2	0 ≤ *T*_*E*_ ≤ 100 day

For each strain, the infection rates $\beta_{s}^{I}$, $\beta_{s}^{C}$, and $\beta_{s}^{A}$ denote the probability of transmitting disease from *I*_*s*_, *C*_*s*_, or *A*_*s*_ to *S*, respectively. On the other hand, as Fig 1 shows, the outcome of each exposed person *E*_*s*_ could be *A*_*s*_, *I*_*s*_, *C*_*s*_, and *D*_*s*_ with the probabilities of $P_{s}^{A}$, $\left(1-P_{s}^{A}\right)\left(1-P_{s}^{C}\right)$, $\left(1-P_{s}^{A}\right)P_{s}^{C}\left(1-P_{s}^{D}\right)$, and $\left(1-P_{s}^{A}\right)P_{s}^{C}P_{s}^{D}$, respectively. By using the method of next-generation matrices [18], we can obtain the following expression for the *R*_0_ of each strain

$$\begin{array}{cc} R_{0}^{\left(s\right)} & =P_{s}^{A}\frac{\beta_{s}^{A}}{\gamma_{s}^{A}}+\left(1-P_{s}^{A}\right)\left(1-P_{s}^{C}\right)\frac{\beta_{s}^{I}}{\gamma_{s}^{I}}+\left(1-P_{s}^{A}\right)P_{s}^{C}\left(1-P_{s}^{D}\right)\frac{\beta_{s}^{C}}{\gamma_{s}^{C}}\\ & =P_{s}^{A}R_{0}^{A(s)}+\left(1-P_{s}^{A}\right)\left(1-P_{s}^{C}\right)R_{0}^{I(s)}+\left(1-P_{s}^{A}\right)P_{s}^{C}\left(1-P_{s}^{D}\right)R_{0}^{C(s)}, \end{array}$$ (2)

where $R_{0}^{A(s)}$, $R_{0}^{I(s)}$, and $R_{0}^{C(s)}$ denote the reproduction number of each outcome, and $R_{0}^{\left(s\right)}$ is obtained by their weighted sum. The weight of each outcome is the probability of its occurrence.

The values of different parameters used in the 2-SEICARD model are listed in Table 1. In our modeling, we assume a wild animal population in which there is no isolation and social policy or prevention strategy such as vaccination, reducing clustering, or wearing masks. Hence, we consider that all *I*_*s*_, *C*_*s*_, and *A*_*s*_ outcomes have the same probability of transmission; i.e., $\beta_{s}^{I}=\beta_{s}^{C}=\beta_{s}^{A}$. According to Eq (2), by considering $\beta_{1}^{I}=\beta_{1}^{C}=\beta_{1}^{A}=0.2$ and the values of 0.13, 0.2, and 0.27 for all *β* values of strain 2, we have $R_{0}^{\left(1\right)}=2$ and $R_{0}^{\left(2\right)}=1.3,2,$ and 2.7, respectively.

All simulation results are obtained by running a Python code available from https://github.com/aarahvar2/SEICARD2.

## Results

In this section, we consider fixed parameters for the first strain in the 2-SEICARD model; i.e., $R_{0}^{\left(1\right)}$= 2 and $P_{1}^{A}$= 0.1. Then, the emergent strain with different values of $R_{0}^{\left(2\right)}$ = 1.3, 2, and 2.7 and $P_{2}^{A}$= 0.1, 0.2 and 0.4 emerges at day *T*_*E*_, where 0 ≤ *T*_*E*_ ≤ 100. In all the above scenarios, we study the effect of the emergent strain on the total number of infected cases and the cumulative mortality, as a measure of severity, from the beginning of the epidemic until we reach the endemic steady state. The total number of infected cases is *N*−*S*_∞_, where *S*_∞_ denotes the number of susceptible cases that have not been infected at all when the disease has gone. The cumulative mortality is the cumulative proportion of deaths in the population due to infection.

### Effect of *R*_0_ and *T*_*E*_ on the total number of infections and the dominant strain

**Fig 2:**
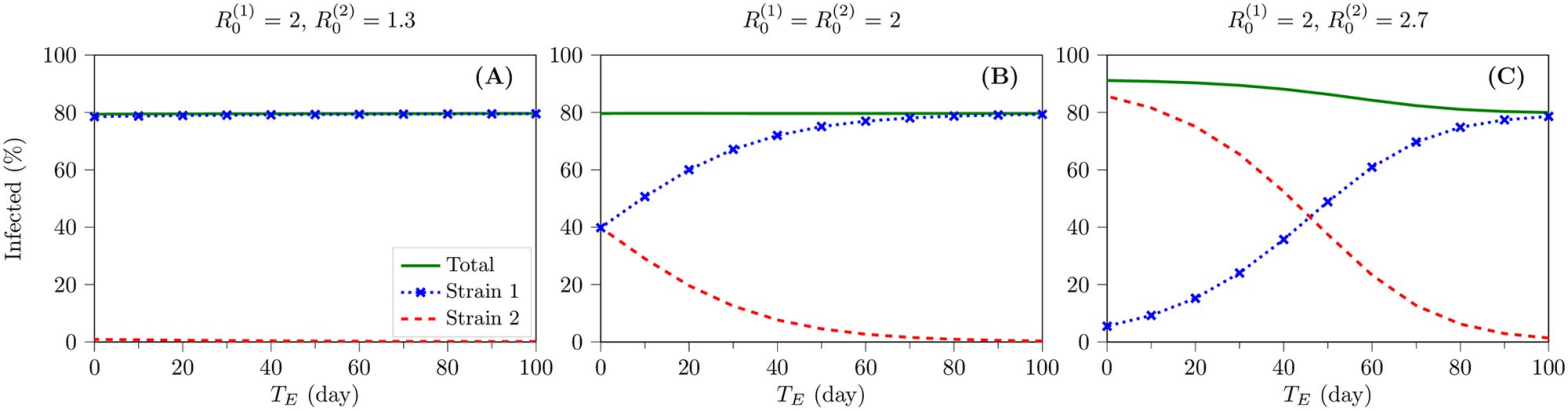
The effect of $R_{0}^{\left(1\right)}$, $R_{0}^{\left(2\right)}$, $P_{1}^{A}$, $P_{2}^{A}$, and *T*_*E*_ on the total number of infected cases in a 2-strain viral epidemic. The fraction of the population who are infected with strains 1 and 2 and the total percentage of the infected cases are depicted for three cases of $R_{0}^{\left(1\right)}>R_{0}^{\left(2\right)}$ (A), $R_{0}^{\left(1\right)}=R_{0}^{\left(2\right)}$ (B), and $R_{0}^{\left(1\right)}<R_{0}^{\left(2\right)}$ (C). Also, these figures demonstrate that the emergence time of the new strain should be considered to determine the winner of the viral competition. In other words, a new, more contingent strain will not be necessarily dominant in the population if it emerges late. The legends of all figures are the same as those in panel A.

The simulation results show that the total number of infected cases does not vary with $P_{2}^{A}$ for the fixed values of $R_{0}^{\left(1\right)}$ and $R_{0}^{\left(2\right)}$. In other words, the values of $R_{0}^{\left(1\right)}$ and $R_{0}^{\left(2\right)}$ determine the total number of infected individuals during the epidemic spread. Provided that $R_{0}^{\left(2\right)}<R_{0}^{\left(1\right)}$, the emergent strain does not have any chance to compete with the primary strain and would become extinct immediately (see Fig 2(A)). Fig 2(B) depicts that in the case of $R_{0}^{\left(2\right)}=R_{0}^{\left(1\right)}$, the total number of infected cases with two strains remains the same as that in the case of spreading only the primary strain in the population with the same value of the basic reproduction number. Moreover, Fig 2(B) demonstrates that for $R_{0}^{\left(2\right)}=R_{0}^{\left(1\right)}$, the total number of infected cases does not vary with the emergence time of the second strain, *T*_*E*_. However, the later emergence of strain 2 results in less proportion of infection with this strain in the population. In contrast, Fig 2(C) depicts that the emergence of a more contagious strain ($R_{0}^{\left(2\right)}>R_{0}^{\left(1\right)}$) increases the total number of infected cases compared to the existence of only the primary strain. Furthermore, Fig 2(C) shows that the new, more contagious strain with larger value of *R*_0_ does not necessarily dominate in the population for the late emergence time.

### Effect of emergent strain on cumulative mortality

**Fig 3:**
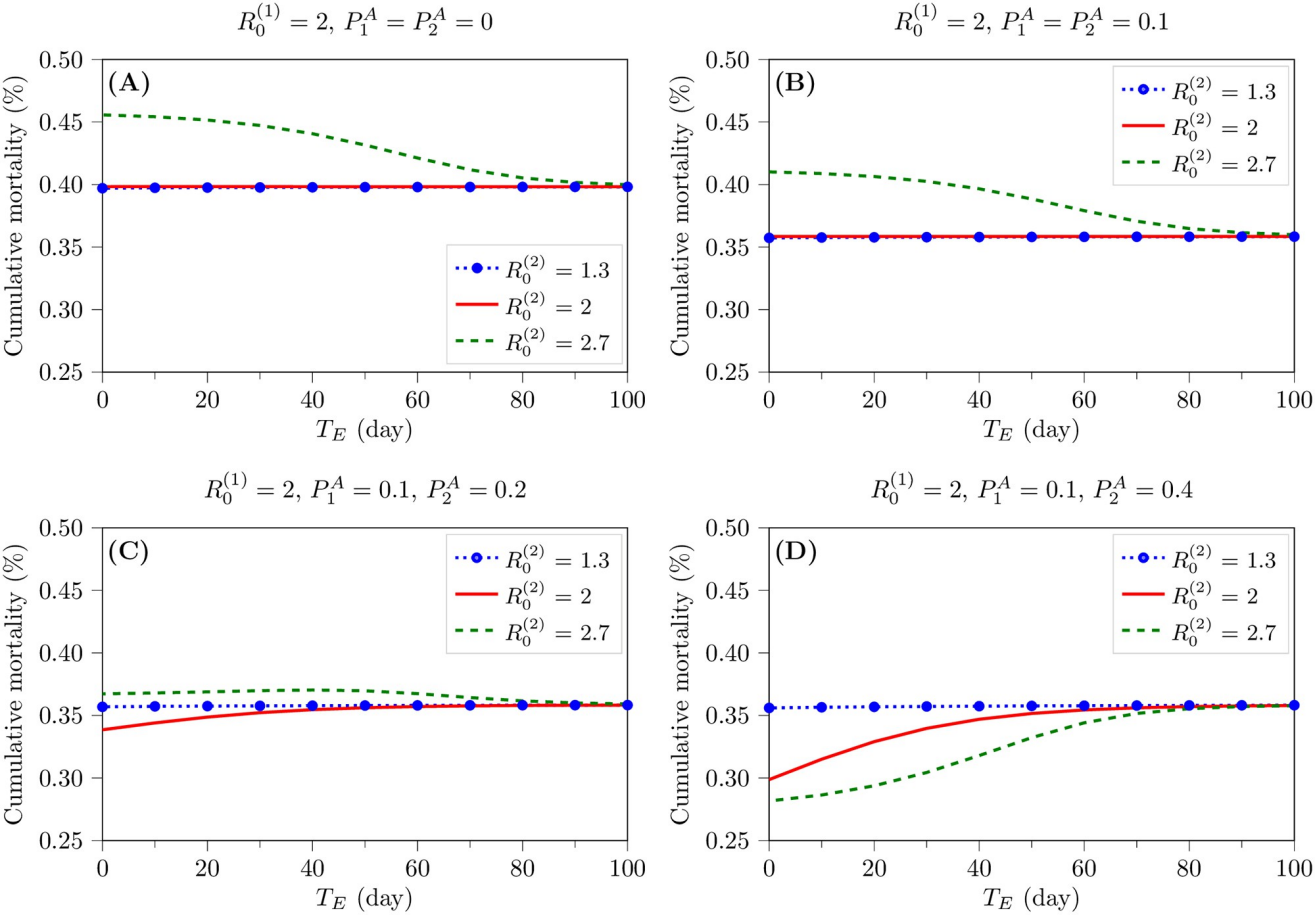
The effect of $R_{0}^{\left(1\right)}$, $R_{0}^{\left(2\right)}$, $P_{1}^{A}$, $P_{2}^{A}$, and *T*_*E*_ on the cumulative mortality. These figures indicate that in the presence of asymptomatic transmission, the emergence of a more-transmissible strain does not necessarily reflect more disease severity. A) The cumulative mortality vs. *T*_*E*_ for the case of all-symptomatic infections in the population for different levels of transmissibility of the new variant ($R_{0}^{\left(2\right)}$ = 1.3, 2, and 2.7). In this case, the cumulative mortality is higher than the case of having asymptomatic infection. B-D) For $R_{0}^{\left(1\right)}=2$ and $P_{1}^{A}=0.1$, these figures show the effect of increase in the fraction of asymptomatic cases of the emergent strain ($P_{2}^{A}$ = 0.1 (B), 0.2 (C), and 0.4 (D)), on the cumulative mortality. The increase in the fraction of asymptomatic cases can reduce the cumulative mortality.

Here, we study the impact of the emergence of a new strain on the disease severity. We used the cumulative mortality as a measure of severity in this study. Fig 3 shows the cumulative mortality in different circumstances. These figures show concurrently the effect of the fraction of asymptomatic individuals infected with strain 2, $P_{2}^{A}$, and the emergence time of the second strain, *T*_*E*_, on the cumulative mortality for $R_{0}^{\left(1\right)}$= 2 and $R_{0}^{\left(2\right)}=1.3,2,$ and 2.7. Fig 3(A) depicts the results of a scenario that all infected cases show symptoms; i.e., there is no asymptomatic infection. However, in Fig 3B–3D, a fraction of population are asymptomatically infected.

As we have discussed, if $R_{0}^{\left(2\right)}<R_{0}^{\left(1\right)}$, the emergent strain cannot compete with the primary strain, and hence, the cumulative mortality remains fixed for all values of $P_{2}^{A}$, equal to the cumulative mortality of the primary strain alone. Hence, in these figures, although blue curves are corresponding to the 2-strain scenario, they also represent the cumulative mortality of the primary strain alone.

Provided that $R_{0}^{\left(1\right)}=R_{0}^{\left(2\right)}$ and $P_{1}^{A}=P_{2}^{A}$, the cumulative mortality remains the same as that of the primary strain alone. On the other hand, as Fig 3(A) and 3(B) depict, if both strains have a similar proportion of asymptomatic cases, i.e., $P_{1}^{A}=P_{2}^{A}$, the cumulative mortality increases with the emergence of a more contagious strain ($R_{0}^{\left(2\right)}>R_{0}^{\left(1\right)}$). In this case, the sooner that the new strain emerges, the more the cumulative mortality increases. By comparing the case of all symptomatic infection (Fig 3(A)) and the case of asymptomatic transmission in the population (Fig 3(B)) we see that the existence of the asymptomatic infection reduces the cumulative mortality. On the other hand, as Fig 3(B) to 3(D) show, in the case of spreading asymptomatic infections, the increase in $P_{2}^{A}$ can reduce the cumulative mortality for $R_{0}^{\left(2\right)}\geq R_{0}^{\left(1\right)}$. For large values of $P_{2}^{A}$, although a more contagious strain emerges, it can make the cumulative mortality less than that before the emergence (Fig 3(D)). Interestingly, for large values of $P_{2}^{A}$, the emergent strain with a higher *R*_0_ decreases the cumulative mortality more.

## Discussion

In this work, we used a 2-strain extension of the classic SEIR epidemic model to study the impact of the emergence of a new, more transmissible strain on a viral epidemic. Unlike many multi-strain models, this model does not assume that two viral strains coexist from the beginning of the epidemic and considers the impact of the emergence time of the new strain on the total number of infections, disease severity, and the winner of the viral competition. In this model, like a wild animal population, no isolation and prevention strategy is adopted in the population. These social policies can be easily added to the model to investigate the viral behavior in human communities.

Our first question was that in the case of a multi-strain viral epidemic, whether an emergent strain with the largest value of *R*_0_, among all available strains in the population, becomes dominant or not. The results of our modeling showed that the emergence of a more contagious strain increases the total number of infected cases. However, being a more contagious strain with the largest value of *R*_0_ does not mean that this strain will be necessarily the winner of the viral competition and the dominant strain in the population. Indeed, besides the values of *R*_0_, the emergence time *T*_*E*_ also determines whether the new strain with more transmissibility dominates in the population or not. In a 2-strain scenario, the sooner emergence of the new, more contagious variant can make it dominant; otherwise, the primary strain remains dominant in the population.

Generally, *R*_0_ is not a measure of the infectious disease severity [19]. However, as we discussed above, the emergence of a more contagious variant increases the number of infected cases. Due to the psychological atmosphere of an epidemic or a pandemic, people intuitively expect that the increase in the number of infected cases results in more disease severity, such as hospitalization, intensive care unit (ICU) admission rate, and mortality. Hence, our second question was that in the case of having a new, more contagious strain, whether or not more infected cases increase similarly the disease severity. To answer this question, we need to consider two different scenarios: i) all infected cases are symptomatic; ii) a fraction of the population are asymptomatically infected. In the first scenario, all infected cases show symptoms, and they can potentially end in the critically infected (C) state. Consequently, the ultimate outcome of a symptomatic patient can potentially be death. However, in the second scenario, the asymptomatic cases recover from infection without being in the critically infected state and ending in death. The results of our 2-strain model confirmed that the existence of asymptomatic transmission in the population can reduce the overall disease severity; i.e., the cumulative mortality reduced by the presence of asymptomatically infected cases in the population compared to the scenario that all infected cases are symptomatic. The results also demonstrated that if the emergent strain with a higher *R*_0_ can infect more fraction of the population asymptomatically, it will decrease the cumulative mortality more. For large values of $P_{2}^{A}$ in a 2-strain scenario, the emergence of the more contagious strain can even make the cumulative mortality less than that before the emergence. Indeed, more transmissibility does not necessarily reflect more severity; i.e., both $R_{0}^{\left(s\right)}$ and $P_{s}^{A}$ values should be considered to correctly determine the effect of the new, more transmissible strain on the viral disease severity.

The results of the study [17] confirms that the above factors, i.e., *R*_0_ and the proportion of asymptomatic transmissions, are the main factors in controlling an infectious disease outbreak. In addition to these factors, our results demonstrated that the emergence time of the new strain is also a crucial factor in a multi-strain viral disease epidemic.

To study the direct impact of the investigated factors, we assumed a wild animal population in which there is no isolation and social policy or restriction. However, in a human population with social policies and restrictions against the emerging variant, we may have a different impact of the new strain. For example, if we limit the spread of the emerging strain by lockdown, the new variant, which potentially can reduce the disease severity, will be less effective. On the other hand, concurrent with the emergence of the new strain, it is challenging to estimate *R*_0_ and the proportion of asymptomatic infections accurately. In this situation, although we know that the new, more contagious strain can potentially impact the disease severity positively, without the accurate estimate of the effecting factors, we cannot optimistically allow the new variant to spread in the population. However, after a while that we can have better estimates, we can decide more effectively about the social restrictions against the emerging strain. Although estimating the proportion of asymptomatic infections could be challenging; however, it is achievable by contact tracing and mass screening during an outbreak of a novel infectious virus [17].

## Conclusion

In this study, we investigated the impact of the asymptomatic transmission and the emergence time of the new, more contagious viral strain on the disease prevalence, disease severity, and the dominant variant in an epidemic. Our results demonstrated that being an emergent strain with more transmissibility, i.e., having a larger basic reproduction number *R*_0_, compared to previous variants, does not necessarily lead to more severity or mean that the new variant will dominate in the population. Indeed, when a new strain with a larger basic reproduction number emerges, it will increase the number of infected cases in the population. However, the creation of more severe outcomes depends on the fraction of asymptomatic transmissions. If a large proportion of infections, due to the new variant, do not show any symptom, they can even reduce the cumulative mortality in the population. Moreover, provided that the emergence time of the new strain is closer to the start of the epidemic, the new, more contagious variant has more chance to win the viral competition and be the dominant strain; otherwise, despite being more contagious, it cannot dominate previous strains.

## Supporting information

S1 File The Python code simulating the results presented in this study. (PY)
